# Preclinical evaluation of high-dose Griffithsin carrageenan fast-dissolving insert for HIV, HSV-2, and HPV prevention

**DOI:** 10.1128/spectrum.01966-25

**Published:** 2026-04-20

**Authors:** J. A. Fernández Romero, P. Barnable, M. L. Visciano, M. Aravantinou, A. Meyer, S. Grecky, F. Schiro, B. Grasperge, J. Gadsden, N. Kommineni, P. Angsantikul, N. Kumar, J. Nuttall, L. B. Haddad, N. Teleshova

**Affiliations:** 1 Science Department, Borough of Manhattan Community College, The City University of New York, New York, New York, USA; 2 Center for Biomedical Research, Population Council, New York, New York, USA; 3 NIH-Maximizing Access to Research Careers at Brooklyn College, New York, New York, USA; 4 Tulane National Primate Research Center 101404 , Covington, Louisiana, USA; Barnard College, Columbia University, New York, New York, USA

**Keywords:** Griffithsin, HSV-2, fast-dissolving insert, HPV, HIV

## Abstract

Griffithsin (GRFT) is an antiviral lectin that blocks human immunodeficiency virus (HIV) entry. When combined with carrageenan (CG), GRFT has strong activity against herpes simplex virus type 2 (HSV-2) and human papillomavirus (HPV). We conducted a preclinical evaluation of a high-dose vaginal fast-dissolving insert (FDI) containing 3 mg GRFT—equivalent to 3× the clinical dose—and compared it with a 1 mg formulation. Studies included FDI disintegration (macaques, rabbits); GRFT pharmacokinetics, pharmacodynamics, and inflammatory response (macaques); toxicity (rats); and immunogenicity (macaques, rats). Disintegration times varied between species, emphasizing the importance of evaluating this parameter in humans. In macaques, cervico-vaginal lavage (CVL) GRFT concentrations exceeded levels associated with *in vivo* efficacy 12 h after administration of the 3 mg GRFT/CG FDI. Potent anti-HIV1_BaL_ activity was detected in CVLs collected 24 h following the 3 mg dose and 12 h after the 1 mg dose. CVLs inhibited HSV-2 at 4 h and HPV at 12 h after both GRFT doses. GRFT/CG FDIs caused no inflammatory response and did not alter vaginal pH. In rats, repeated daily dosing at up to 10× the clinical dose revealed no safety concerns. Although anti-drug antibodies (ADAs) developed in macaques and rats, systemic GRFT was undetectable, suggesting that ADAs are unlikely to alter pharmacokinetics. Overall, the CVL GRFT concentrations and anti-HIV activity following administration of 3 mg GRFT/CG FDIs suggest a longer protection window—at least 12 h—against HIV-1 compared with the 1 mg FDI, and both formulations demonstrate activity against HSV-2 and HPV. These findings support continued GRFT/CG FDI development.

**IMPORTANCE:**

There is an undeniable need to develop multipurpose prevention products that simultaneously protect against human immunodeficiency virus (HIV) and other sexually transmitted pathogens. To that end, we conducted a preclinical evaluation of a carrageenan-based fast-dissolving vaginal insert containing 3 mg of anti-viral lectin Griffithsin—equivalent to 3× clinical dose—and compared it with a 1 mg insert. Both formulations were safe and demonstrated potent anti-HIV, herpes simplex virus type 2 (HSV-2), and human papillomavirus (HPV) activity. Our data suggest that a 3× clinical dose Griffithsin formulation may afford an extended window of protection against HIV compared to the 1× dose formulation. These data support further development and clinical testing of high-dose Griffithsin carrageenan fast-dissolving vaginal insert.

## INTRODUCTION

The global burden of sexually transmitted infections (STIs) is significant, including 1.3 million new human immunodeficiency virus (HIV) infections reported in 2024 (1). Other STIs, such as genital herpes simplex virus (HSV) and human papillomavirus (HPV), are known to increase HIV acquisition and are currently rising in prevalence (2–4).

Pre-exposure prophylaxis (PrEP) remains the cornerstone of HIV prevention, which includes oral (tenofovir disoproxil fumarate/emtricitabine [TDF/FTC] and tenofovir alafenamide/emtricitabine [TAF/FTC]) (5, 6), and long-acting injectable (cabotegravir long-acting and lenacapavir) (7, 8) PrEP, and dapivirine vaginal ring (9). Lenacapavir has demonstrated unprecedented efficacy in Phase 3 trials, conferring 100% protection in cisgender women and a 96% reduction in HIV incidence among cisgender men and gender-diverse populations (10–12). Despite these major advances, critical gaps in HIV prevention persist, including concerns of antiretroviral resistance, the lack of on-demand and over-the-counter prevention options, and the continued reliance on HIV testing prior to PrEP initiation.

To date, there is no preventive or therapeutic vaccine against HSV (13–15), making control of HSV transmission a challenge. Although multiple vaccine candidates have demonstrated the ability to stimulate an immune response in early-phase trials, none have yet been approved for clinical use. In contrast, prophylactic HPV vaccines are among the most effective cancer prevention interventions in modern medicine and are designed to prevent infection with oncogenic HPV types responsible for cervical, anogenital, and oropharyngeal cancers. Although ~90% coverage of oncogenic HPV types is broad, the vaccines do not cover all high-risk HPV types. Moreover, vaccine efficacy is highly dependent on timing; HPV vaccines are most effective when administered prior to sexual debut. HPV-16/18 prevalence was reported to be 89% lower among individuals vaccinated before sexual debut, but only 41% lower among those vaccinated after sexual debut compared with the unvaccinated individuals (16). In addition, several barriers to global HPV vaccine uptake exist, including cost and cold-chain requirements (17).

At present, there are no approved products that provide broad-spectrum protection against multiple STI pathogens. Consequently, there is a critical need for multipurpose prevention technologies (MPTs) capable of simultaneously protecting against HIV and other sexually transmitted pathogens, such as HSV and HPV (18–21). To address this unmet need, the Population Council is developing a user-controlled, on-demand MPT product—vaginal Griffithsin (GRFT) fast-dissolving insert (FDI)—to protect against HIV, HSV-2, and HPV.

On-demand products provide several advantages, including adherence flexibility, cost-effectiveness, and accessibility to an inclusive approach to HIV prevention that accommodates diverse needs and preferences (19). The FDI is a discreet, easy-to-use, and portable dosage form that has been endorsed by end-users as a highly desired formulation (19).

GRFT is a potent anti-viral lectin that blocks HIV entry at picomolar concentrations (22–24). GRFT’s high affinity for HIV-1 gp120 and its unique mode of action against HIV (25, 26) gives this molecule an important advantage due to lack of cross-resistance to antiretroviral (ARV) drugs currently used for HIV treatment and prevention. This protein has shown no toxicity in various animal models (27–29) and remains active under a variety of conditions, including different pH and temperatures (30).

GRFT was shown to inhibit HSV-2 entry without affecting initial viral attachment to the cell surface, indicating action at a post-binding step. Binding of GRFT to HSV-2 glycoprotein D (gD) interferes with gD-mediated interactions with cellular entry receptors, thereby preventing membrane fusion and viral entry. Anti-HPV activity of GRFT is mediated through targeting the secondary HPV receptor α6 and inducing its internalization. When combined with carrageenan (CG), GRFT has greater activity against HSV-2 and HPV (31).

We previously reported that a first-in-human Phase 1 study of 4 mg GRFT in a CG-based vaginal gel demonstrated no systemic absorption and no safety signals in healthy, HIV-negative women (32). We also demonstrated high GRFT concentrations in Rhesus macaque vaginal fluids in non-Depo-Provera treated animals, as well as in Depo-Provera treated animals at 4 and at 8 h post-administration of 1 mg GRFT/CG FDI (33), equivalent to 1× the clinical gel dose (4 mg). One milligram GRFT freeze-dried FDI protected Rhesus macaques (2/10 infected macaques in the treatment group vs 10/10 in the placebo group) from a high-dose vaginal SHIV SF162P3 challenge 4 h after FDI insertion (33).

In the present study, we conducted a preclinical evaluation of GRFT carrageenan (GRFT/CG) FDI containing a GRFT dose equivalent to 3× that of a previously tested clinical gel dose (32) and compared it with a dose equivalent to 1× the clinical dose in different preclinical models. Our goal was to perform exploratory *in vivo* (disintegration time, mucosal safety*,* plasma PK, and vaginal GRFT concentrations, immunogenicity) and *ex vivo* (window of protection against HIV, HSV, and HPV) FDI evaluation in Rhesus macaques. We previously conducted a 14-day GRFT intravenous study in Sprague-Dawley rats, a 14-day intravaginal study in rats, and a 14-day intravaginal study in New Zealand white rabbits. These studies did not reveal safety concerns (33). To further support the development of the GRFT/CG FDI, herein we conducted an FDA-required 28-day intravaginal rat toxicity and exploratory immunogenicity study using up to 10× the proposed maximum clinical dose. There is currently no optimal model to evaluate the disintegration of vaginal inserts or tablets *in vivo* (34). Therefore, we evaluated FDI disintegration in macaques and rabbits.

## RESULTS

### The 3 and 1 mg GRFT/CG FDIs demonstrate different disintegration profiles in macaques

**Fig 1:**
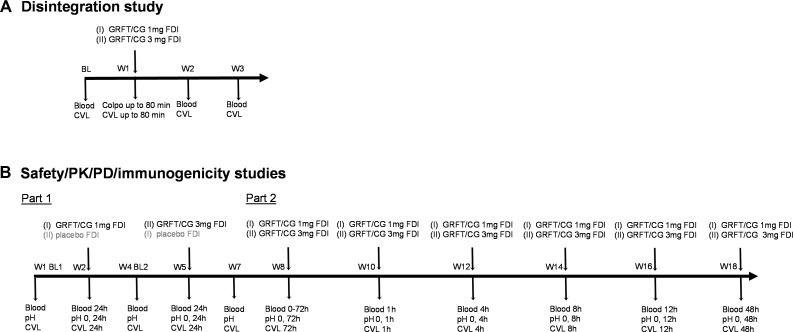
Rhesus macaque studies timeline and biological sample collection points. Macaques were assigned to two groups (I and II, *n* = 6 each), with group I receiving 1 mg GRFT/CG FDI (or placebo) and group II receiving 3 mg GRFT/CG FDI (or placebo) throughout. Studies were performed to determine the disintegration profile of FDIs (**A**), followed by safety/PK/PD/immunogenicity evaluation (**B**), with 4 weeks between the studies. EDTA blood and cervico-vaginal lavages (CVLs) were shipped overnight and processed for plasma and clarified CVL, respectively. pH measurements were taken immediately prior to FDI administration and at the indicated time points. W, week; BL, baseline.

FDI disintegration was analyzed following administration of 1 and 3 mg GRFT/CG FDIs (six animals per group) (Fig. 1). One milligram GRFT/CG FDIs disintegrated faster than 3 mg GRFT/CG FDIs (Fig. S1). One milligram GRFT/CG FDIs dissolved or became amorphous after 20 min (*n* = 2) or 60 min (*n* = 4). One out of four animals with FDIs dissolving at 60 min exhibited menstrual blood. The 3 mg GRFT/CG FDI completely dissolved in only two animals (*n* = 1 at 40 min, *n* = 1 at 80 min); the other four FDIs were decreased in size and appeared to be in the process of dissolving when colposcopy was concluded. One of these four animals exhibited menstrual blood.

### A 3 mg GRFT/CG FDI releases high concentrations of GRFT in macaques over a longer period than a 1 mg GRFT/CG FDI

**Fig 2:**
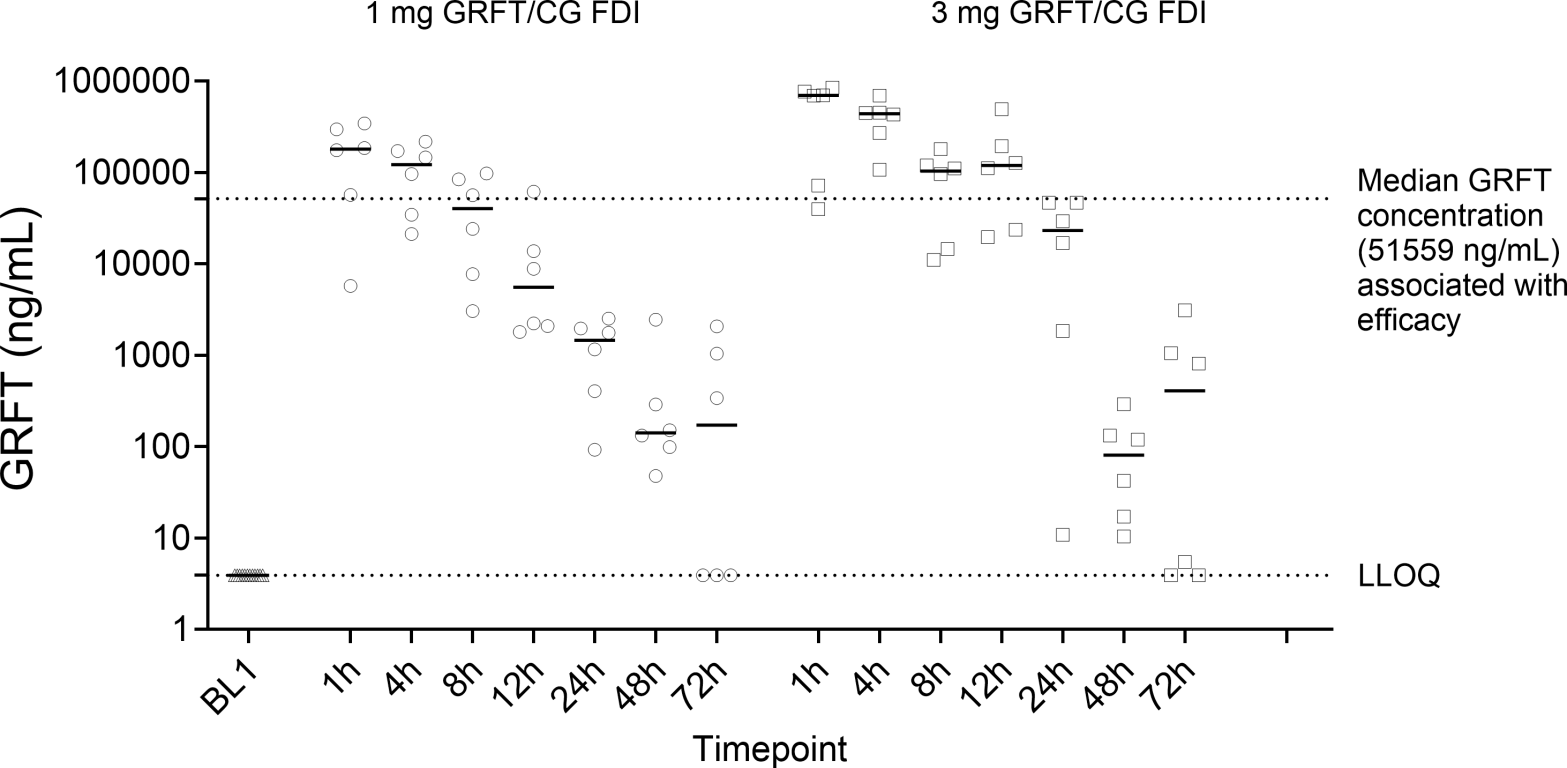
CVL GRFT concentrations. Each symbol represents an individual macaque. Horizontal solid lines show median concentrations.

To determine vaginal and plasma GRFT concentrations at multiple time points post-FDI insertion, animals were administered FDIs every 2 weeks. Using samples collected during the disintegration study (Fig. 1), we confirmed that no residual GRFT was detected 2 weeks following FDI administration. Expectedly, higher cervico-vaginal lavage (CVL) GRFT concentrations were detected after 3 mg GRFT/CG FDI administration vs the 1 mg GRFT/CG FDI (Fig. 2). GRFT concentrations 1–12 h after 3 mg GRFT/CG FDI insertion were higher than concentrations associated with efficacy against high-dose SHIV162P3 challenge (33). Unsurprisingly, substantial variability in measured GRFT concentrations was observed at 72 h after FDI insertion, likely reflecting differences in GRFT clearance rates among the animals. Plasma GRFT concentrations were below the lower limit of quantification at all time points (1, 4, 8, 12, 24, 48, and 72 h) after FDI administration.

### GRFT in CVL is effective against HIV-1_BaL_, HSV-2, and HPV infection *ex vivo*

**Fig 3:**
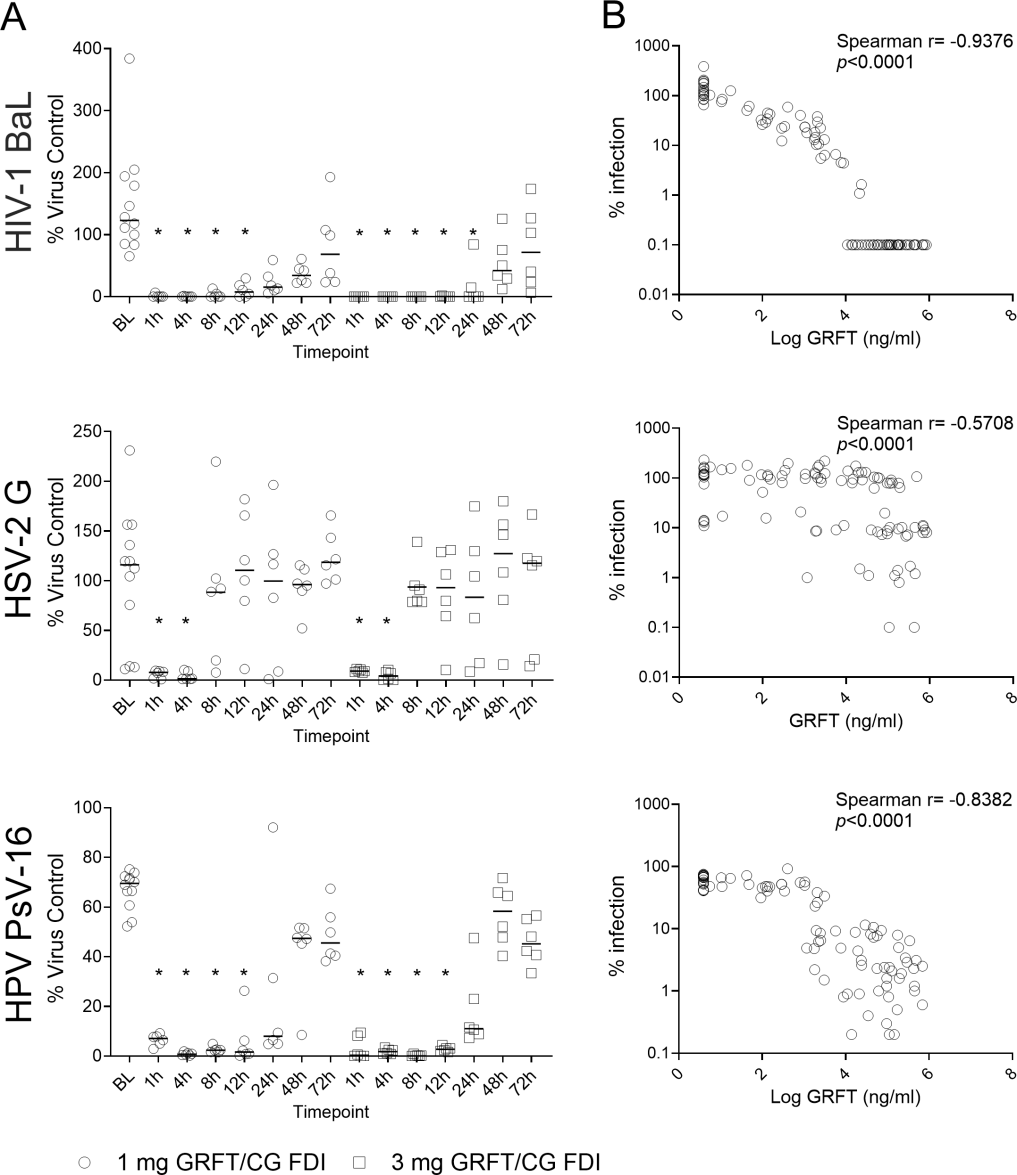
Anti-viral activity of CVL and GRFT PK/PD relationship. (**A**) Shown is the % viral titer for CVLs collected from macaques administered GRFT/CG FDIs. Each symbol represents an individual animal. Horizontal lines show median concentrations. Each sample was tested using two or five replicates, and the mean titer for each condition was compared to the mean titer obtained for baseline CVLs. (**B**) Spearman correlation analysis demonstrates that the higher the concentration of GRFT in CVLs, the more potent the antiviral activity.

CVLs from the 3 mg GRFT/CG FDI group collected up to 24 h post-FDI administration reduced HIV infection in cell-based assays, while those from the 1 mg GRFT/CG FDI group reduced infection at time points up to 12 h post-administration. HPV PsV16 and HSV-2 infection was inhibited at time points up to 12 and 4 h, respectively, in both groups. Anti-viral activity correlated with GRFT concentrations in CVL (Fig. 3). However, as demonstrated in previous studies (31), part of the antiviral activity against HSV-2 and HPV is mediated by CG in the formulation. The lack of a validated method to quantify CG in CVLs did not allow for the establishment of a correlation with the observed antiviral activity.

### FDIs release GRFT without inflammation and do not change vaginal pH in macaques

**Fig 4:**
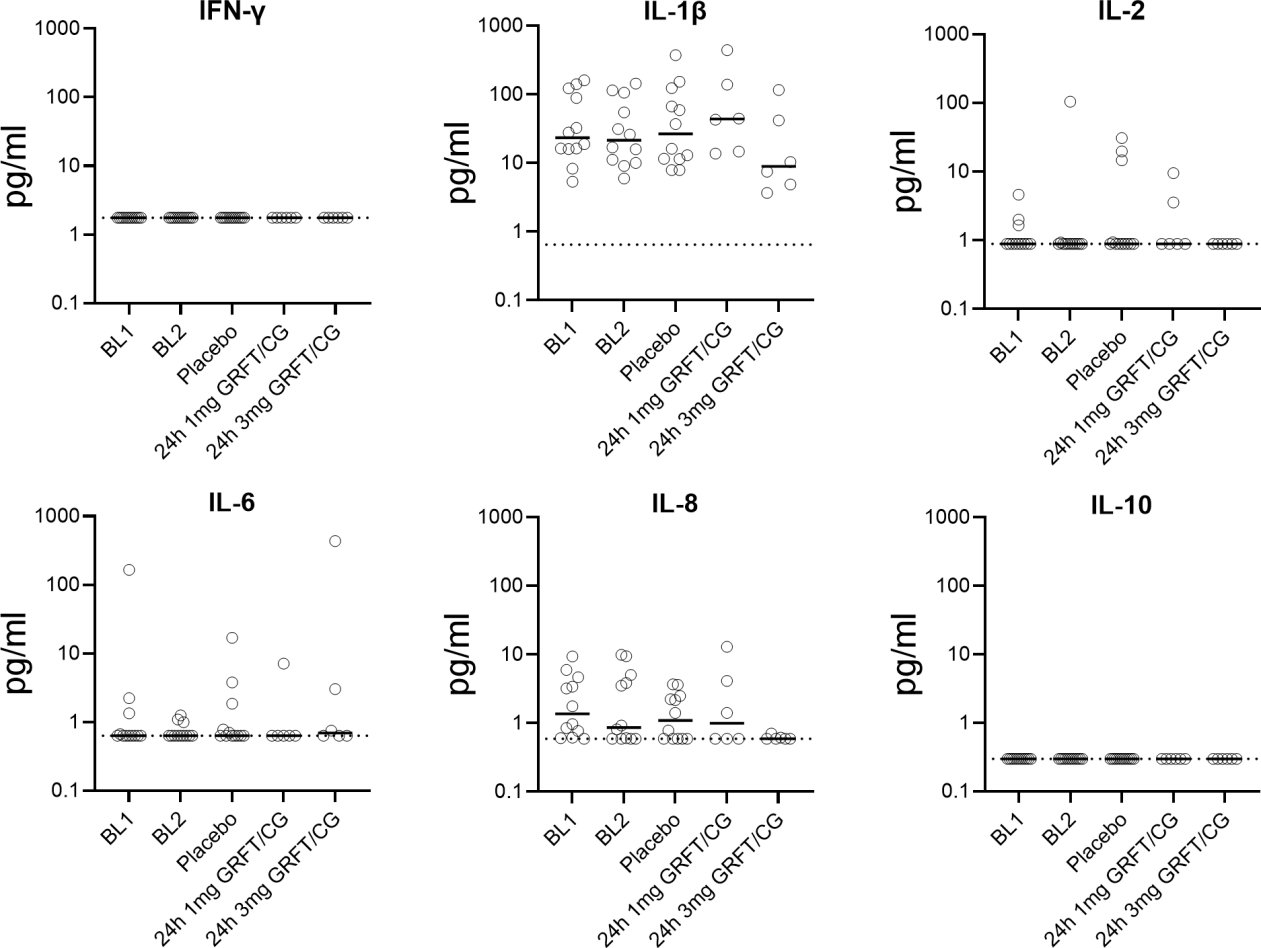
CVL cytokine concentrations after administration of 1 and 3 mg GRFT/CG FDIs. Each symbol represents an individual macaque. Horizontal solid lines show median concentrations. Dotted lines indicate LLOQs.

To test if GRFT/CG FDI administration induces inflammatory responses, cytokine concentrations were measured in CVLs collected 24 h after GRFT/CG FDI administration and compared to those in samples from macaques receiving placebo FDIs. No changes in cytokine concentrations, specifically IFN-γ, IL-1β, IL-2, IL-6, IL-8, and IL-10, were detected following GRFT/CG FDI administration as compared to placebo FDIs or baseline samples collected 1 week before FDI administrations (Fig. 4). Similarly, no changes in vaginal pH were observed at 1–72 h following administration of GRFT/CG FDIs or placebo FDIs (Fig. S2).

### GRFT/CG FDIs induce anti-GRFT antibodies in macaques

**Fig 5:**
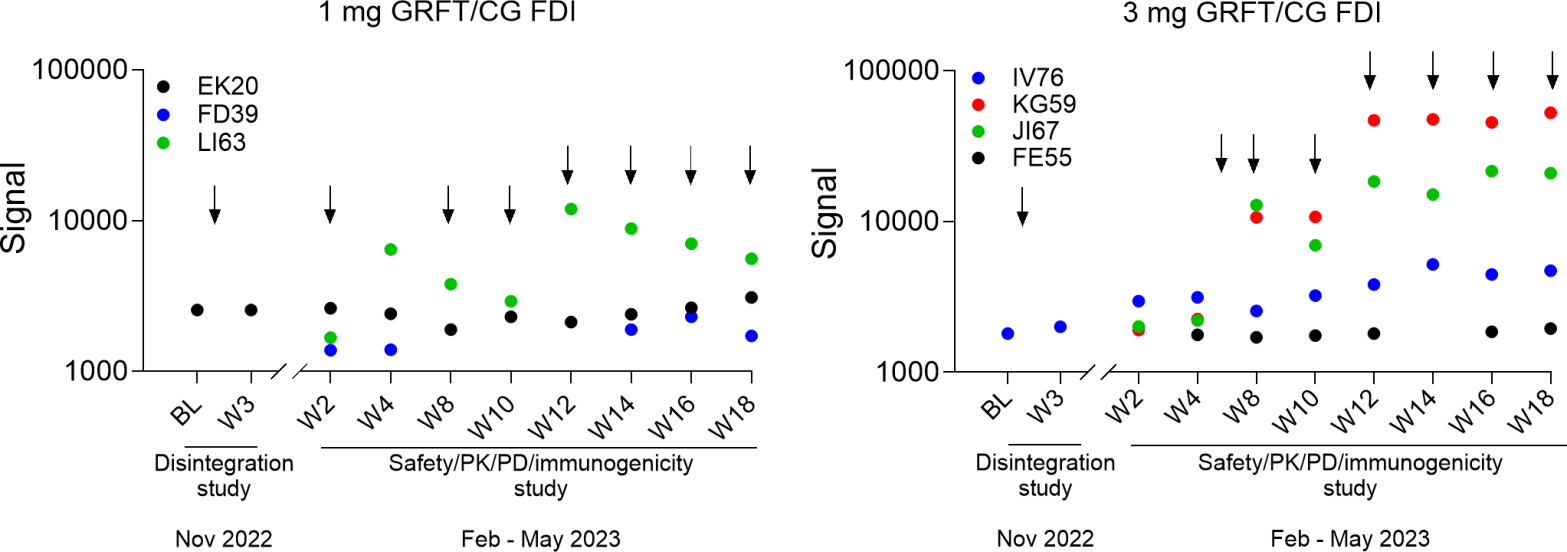
Plasma anti-GRFT antibodies following repeated administrations of 1 and 3 mg GRFT/CG FDIs. Plasma samples from six animals/per group/per time point were analyzed. Shown are RLU values (signal) above assay cut points in individual animals considered ADA positive. Arrows indicate GRFT/CG FDI insertions.

Anti-GRFT anti-drug antibodies (ADAs) were measured in plasma samples collected over the course of the studies. The signal (relative light units; RLU) above assay cut point or above cut point and signal at the baseline was considered positive for ADAs (Fig. 5). ADAs were detected in three animals and in four animals from the 1 and 3 mg GRFT/CG FDI groups, respectively, with higher signal detected at later time points in the 3 mg GRFT/CG FDI group (Fig. 5).

### Fast disintegration of 3 mg GRFT/CG FDIs in rabbits

We conducted a non-GLP FDI disintegration study in rabbits (*n* = 3 animals per time point). FDIs disintegrated completely 20 min after administration in three animals. In a separate group, approximately 50% of the FDI remained in all three animals at 10 min. Reddened areas in the vagina were noted, likely due to trauma during the administration procedure.

### High-dose GRFT is safe in rats

We conducted a GLP 28-day repeat dose toxicity study in which groups of 10 female rats received GRFT/CG as gels with the same composition as the FDI at concentrations of 0, 3, or 5 mg/mL, administered using a dose volume of 0.1 mL/animal, resulting in doses of 0, 0.3, or 0.5 mg/day. The rat was selected because of its common use in toxicology studies and the wealth of background data available on this species. The selected doses are equivalent to 6× and 10× the planned clinical dose and were chosen to establish adequate safety margins to support clinical trials. No evidence of toxicity was observed, and GRFT was undetectable in serum. ADAs were observed at the end of treatment in the serum of two animals (one treated with 3 mg/mL and another treated with 5 mg/mL).

## DISCUSSION

Our macaque data demonstrating CVL GRFT concentrations above those associated with protection against vaginal challenge with SHIV162P3 after administration of 1 mg GRFT/CG FDI (33) suggest a longer window of protection against HIV-1 (12 h or longer) for the 3 mg GRFT/CG FDI. Potent *ex vivo* anti-HIV-1_BaL_ activity of GRFT in CVLs collected up to 24 h post-3 mg GRFT/CG FDI administration also suggests that this higher dose GRFT/CG FDI may afford an extended window of protection compared to the 1 mg dose. Furthermore, our results demonstrate potent activity of both 1 and 3 mg GRFT/CG FDIs against HSV-2 and HPV PsV 16. Although suggestive that the higher dose FDI could offer protection for at least 12 h, clinical studies to evaluate the window of protection after insertion of the FDIs at comparative doses (4 and 12 mg) are needed.

Similar to the 1 mg GRFT/CG FDI results (33), plasma GRFT concentrations were undetectable in macaques and in rats following administration of the high-dose GRFT/CG FDI, indicating no or minimal systemic absorption of GRFT.

Lack of inflammatory response, lack of vaginal pH changes in macaques, and no safety concerns in the 28-day rat study at up to 10× the clinical dose add to the reported safety of GRFT (24, 27–29).

Although GRFT is considered to be a weak immunogen, immunogenicity of biologic drugs is a widely acknowledged issue, and efforts to de-immunize GRFT by structure-guided elimination of T-cell epitopes have been suggested (35, 36) before the product is used in humans. Repeated exposure to GRFT induced ADA responses in macaques and rats in our study. However, since no quantifiable GRFT was observed in plasma, the presence of ADAs (even those with neutralizing activity) would not alter the pharmacokinetic or systemic safety profiles. It should be noted that ADA studies in preclinical species are generally limited in their ability to predict the development of ADA responses to therapeutic protein products in humans, and, therefore, it is critical to also evaluate ADA responses in humans (37).

Our FDI disintegration studies demonstrate variable results in rabbits and macaques, with a rapid process seen in rabbits (within 20 min) and longer (>1 h) disintegration time of high-dose GRFT/CG FDI in macaques. There are known differences between the rabbit, Rhesus macaque, and human reproductive tracts that may contribute to the FDI disintegration process (38). Most of the rabbit vagina is lined by columnar epithelium (39). The rabbit vagina lacks cervical mucus production and *Lactobacillus* spp. and has a pH around 7–8. The Rhesus macaque vagina is lined with stratified keratinized epithelium and has a pH around 6, as reported in reference 40 and in our study. Like in women, Rhesus macaque vaginal epithelium thickness changes during the menstrual cycle; however, Rhesus macaques have greater levels of vaginal keratinization than women (41). The Rhesus macaque vaginal microbiome is similar to a bacterial vaginosis-like microbiome in women, with a low abundance of *Lactobacillus* spp. Proteome characteristics in cervical secretions from Rhesus macaques are similar to those in humans (42). The maximum ambient vaginal fluid volume in humans is believed to be 1–2 mL (43), and how it compares to vaginal fluid volume in Rhesus macaques is not well characterized. It is unclear which differences between the models contributed the most to the discrepant FDI disintegration results. Studies in humans will ultimately inform us on the time required for FDI disintegration *in vivo*. Furthermore, while we anticipate human studies may reveal some variability in disintegration kinetics, protections may be evident prior to full disintegration.

Overall, compared to ARV drugs, GRFT offers several key advantages. First, GRFT exhibits remarkably potent anti-HIV activity, including ARV-resistant HIV strains (22–26), since it targets glycan structures that evolve under strong functional constraint. Second, it also shows activity against HSV-2 and HPV (31). Third, GRFT shows minimal systemic absorption after vaginal administration, reducing potential for systemic toxicity or off-target effects (32, 33). Fourth, it has a potential for over-the-counter use. However, lectin-based antivirals also have disadvantages relative to small molecules. As protein biologics, lectins carry a risk of immunogenicity and ADA formation, as observed in both macaques and rats in this study and discussed above. Additionally, the manufacturing complexity and cost of protein biologics are higher than that of traditional small-molecule ARVs, which could impact scalability and accessibility. Finally, although lectin binding to viral glycans is highly conserved, the long-term evolutionary pressure on viral glycosylation patterns is not yet fully understood.

Taken together, our previously published GRFT/CG FDI safety and efficacy studies, Phase 1 clinical trial data using GRFT vaginal gel (32, 33) and data presented herein indicating safety and extended window of protection against HIV *ex vivo* and activity against HSV and HPV support further development and clinical testing of a high-dose vaginal GRFT/CG FDI for HIV, HSV, and HPV prevention.

## MATERIALS AND METHODS

### Fast-dissolving inserts

GRFT/CG and placebo FDIs were manufactured at the Population Council. GRFT was produced by International Flavors & Fragrances Inc. (IFF, New York, NY) (lot G2). Table 1 details FDI composition. FDIs were manufactured following a previously established method (44). In brief, GRFT, sucrose, mannitol, dextran 40, and carrageenan were sequentially dissolved in water with mixing at 300 rpm by the RW20 overhead stirrer (IKA, Wilmington, NC) to form a homogeneous aqueous gel. The gel was dispensed into PCR microtubes using a positive displacement pipette and lyophilized using a validated lyophilization protocol (44). Placebo FDIs were prepared using the same procedure, omitting the addition of GRFT. The resulting FDIs were stored at 2°C–8°C until use. The bullet-shaped FDIs were 15 mm long, 2.5 mm in diameter at the narrowest point, and 5 mm in diameter at the widest point. GRFT stability for the duration of studies was confirmed using reversed-phase ultra-performance liquid chromatographic (RP-UPLC).

**TABLE 1:** FDI composition

FDI	Composition
Placebo FDIs	Carrageenan: 3 mg Mannitol: 12 mg Dextran 40: 8 mg Sucrose: 2 mg
GRFT/CG FDI	Composition as above, plus 1 or 3 mg GRFT

### NHP studies

Adult female Indian Rhesus macaques (Macaca mulatta) were utilized. Animals ranged in age from 7 years 4 months to 19 years 6 months, and in weight from 6.25 to 12.8 kg at the start of the studies. Macaque studies were carried out at Tulane National Primate Research Center (TNPRC, Covington, LA) in compliance with the regulations stated in the Animal Welfare Act, the Guide for the Care and Use of Laboratory Animals (45, 46), and TNPRC animal care procedures. The TNPRC Institutional Animal Care and Use Committee approved the studies (OLAW Assurance #A4499-01). TNPRC receives full accreditation by the Association for Accreditation of Laboratory Animal Care (AAALAC #000594). FDI disintegration, and safety, PK, PD, and immunogenicity studies were conducted (Fig. 1).

Animals were divided into two groups (Groups I and II, six animals each). Group I: mean age 14.9 years, mean weight 9.14 kg. Group II: mean age 13.7 years, mean weight 8.74 kg. Group I received 1 mg GRFT/CG FDIs or placebo FDIs, and Group II received 3 mg GRFT/CG FDIs or placebo FDIs (Fig. 1).

FDI disintegration was monitored by colposcopy. With the animals positioned in sternal recumbency and using DeBakey forceps, FDIs were manually placed close to the cervix while a vaginal speculum was employed to visualize the vagina and cervix. Colposcopy images were taken using a Karl Storz rigid scope pre-insertion, immediately after insertion, and at 5, 20, 40, 60 min, and up to 80 min post-insertion to observe FDI disintegration. Blood and CVLs were collected 1 and 2 weeks post-insertion to check for residual GRFT.

For CVL collection, 5 mL saline solution was gently infused into the vaginal vault via a sterile 10-mL syringe attached to a sterile pediatric gastric feeding tube (size 8 French) of adjusted length, and CVL fluid was drawn out with the same device. Blood and CVL samples were transported overnight at 4°C to the Population Council’s Center for Biomedical Research. Plasma and CVL samples were stored at −80°C for further analysis.

For the inflammatory response evaluation, GRFT/CG FDIs or placebo FDIs were administered, and CVL and blood were collected 24 h post-insertion.

Plasma and CVL GRFT concentrations were determined through repeated single administrations of the FDIs at 2-week intervals, with CVLs collected at the indicated time points. Plasma PK was determined over 72 h following a single FDI insertion.

Vaginal secretions were collected using a swab, then rolled on a pH indicator strip to measure vaginal pH.

### GRFT assay in macaque plasma and CVL

The plasma and CVL concentrations of GRFT after *in vivo* exposure were determined through MesoScale Diagnostics (MSD, Rockville, MD) electrochemiluminescent immunoassay (MSD-ECL) at the Population Council. Briefly, a streptavidin-coated plate was incubated at room temperature with biotinylated mouse anti-QGRFT monoclonal antibodies (Bt-mAb; Green Mountain Antibodies, Burlington, VT; and biotinylation was performed by KCAS Bio) for 1 h. Following incubation, the plate was washed and blocked with Pierce Protein-Free Buffer (ThermoFisher Scientific, Waltham, MA) to prevent non-specific binding. After incubation, the plate was washed again, and Standards, QCs, and study samples were diluted 1:100 (minimal required dilution) in buffer prior to being added to the assay plate. After sample incubation at room temperature, the plate was washed, and a rabbit anti-QGRFT pAb detection antibody was added to the plate. After primary detection antibody incubation at room temperature, the plate was washed again, and a Sulfo-Tag goat anti-rabbit IgG detection antibody was added to the plate. The assay was incubated at room temperature before undergoing a final wash step. A 2× Read T Buffer was added, and the MSD plate was read using Methodical Mind software. This method was previously validated over the range of 3.91 to 500 ng/mL. Data were collected using the QuickPlex 120MM utilizing acquisition software Methodical Mind v1.0.38 (MSD). The data from Methodical Mind were entered into GraphPad Prism v10 software (GraphPad, Boston, MA). Sample concentrations of GRFT were interpolated from a standard curve generated with known concentrations of GRFT.

### Anti-drug antibody assay in macaque plasma

ADA concentrations were analyzed at the Population Council using the MSD-ECL assay that utilizes the bivalent binding capability of anti-GRFT antibodies to form a bridging complex with biotinylated GRFT and ruthenylated GRFT to generate RLU for the measurement of anti-GRFT antibodies in the matrix. Detection of the anti-drug antibodies was done using a screening approach with a cut-point established to support a 5% false-positive rate. Assay Master Mix was prepared by diluting biotinylated QGRFT (B-QGRFT; KCAS) and ruthenylated QGRFT (R-QGRFT; KCAS) in assay diluent buffer. A 2× stock of Master Mix was added in equal volume to the samples, positive and negative controls, and incubated for 90 min to allow for immune complex formation. Concurrently, the MSD streptavidin plate was blocked for 1 h to prevent non-specific binding. Following incubation, the plate was washed, and then samples and controls were added to the plate and incubated for 1 h. Finally, the plate was washed, then 2× Read Buffer was added, and the plate was read promptly on the MSD QuickPlex SQ 120MM. Analysis of the signal generated through Methodical Mind software was performed using Excel (Microsoft, Redmond, WA). If the RLU response was greater than the screening cut point (GeoMean multiplied by the Tier I Factor of 1.3 [sample signal>(1.3×GeoMean)]), then the sample was reported as positive for anti-GRFT antibodies.

### Cytokine and chemokine assay in macaque CVL

Clarified CVLs from baseline and 24 h post-FDI insertion were analyzed at the Population Council using the V-Plex Proinflammatory Panel 1 NHP kit (MSD) according to the manufacturer’s protocol. The analytes measured were IFN-γ (LLOQ 1.76 pg/mL), IL-1β (LLOQ 0.646 pg/mL), IL-2 (LLOQ 0.89 pg/mL), IL-6 (LLOQ 0.633 pg/mL), IL-8 (LLOQ 0.591 pg/mL), and IL-10 (LLOQ 0.298 pg/mL). ECL was measured on the QuickPlex SQ120MM, and data were analyzed using MSD Discovery Workbench 4.0 software.

### Anti-viral activity in macaque CVLs

Anti-HIV-1_BaL_ activity was tested in TZM-bl cells, using the MAGI assay as we previously published (47). HIV-1_BaL_ was generated in CD8-depleted human PBMCs stimulated with IL-2 and PHA (48). Anti-HSV-2 and anti-HPV PsV-16 activity was tested in Vero cells and in HeLa cells, respectively, as we previously published (31, 49). In each assay, the virus (30 µL) was incubated in saline (virus control; 70 µL) or with each CVL sample (70 µL) for 30 min at 37°C, 5% CO_2_, and 98% humidity. After incubation, the virus was titered using MAGI assay (two replicates), pseudoviral assay (five replicates), and plaque assay (two replicates) for HIV-1_BaL_, HPV PsV-16, and HSV-2 G strain, respectively. The percent of viral titer compared to the virus control was calculated for each data point.

### Statistics

Data were analyzed using GraphPad Prism v10 software to perform the Kruskal-Wallis test with Dunn’s multiple comparisons test (*P < 0.05).

### Non-GLP disintegration study in rabbits

The study was conducted at Charles River Laboratories (Ashland, OH, USA). Six female New Zealand White Rabbits received a single FDI containing 3 mg GRFT by intravaginal administration. The FDI was administered with the rabbit in dorsal recumbency or upside down using a rubber cannula (28 Fr, at least 10 cm in length), which was placed into the end of the rubber catheter and inserted 6 to 8 cm into the vagina. External lubricant was applied to the cannula, taking care to ensure the lubricant did not come into contact with the FDI. Three rabbits were euthanized at 10 min post-dosing, and the other three rabbits were euthanized at 20 min post-dosing by intravenous injection of sodium pentobarbital, followed by exsanguination. At necropsy, a macroscopic examination was performed that included evaluation of the vagina and an assessment of the disintegration of the FDI. The FDI status was characterized as either (i) FDI intact, (ii) partial disintegration (FDI > 50% intact), (iii) partial disintegration (FDI ≤ 50% intact), or (iv) disintegration complete (only gel present—no particles of FDI).

### GRFT-FDI gels and placebo gels

The gels were manufactured at the Population Council. GRFT lot G2, manufactured using *Bacillus subtilis*, was produced by IFF. GRFT lot CMB-BSD-0900-001, manufactured using *Nicotiana benthamiana*, was provided by the Population Council. GRFT, sucrose, mannitol, dextran 40, and carrageenan were sequentially added to water and mixed at 300 rpm by the RW20 overhead stirrer (IKA, Wilmington, NC) to form a homogeneous aqueous gel. The resulting GRFT-FDI gels were then dispensed into plastic containers and stored at −20°C until further use. Vehicle gel samples were prepared using the same method, excluding the addition of GRFT. GRFT stability for the duration of studies was confirmed using RP-UPLC.

### Twenty-eight-day GLP local and general toxicity study in rats

This study was conducted at Charles River Laboratories. Groups of 10 female Sprague Dawley rats received gels containing GRFT manufactured either via *Bacillus subtilis* or *Nicotiana benthamiana* for 28 consecutive days, as presented in Table 2.

**TABLE 2:** Twenty-eight-day GLP local and general toxicity study in rats^*a*^

Group no.	Test article	Dose concentration (mg/mL)	Dose volume (mL/animal)	No. of animals	
				Main study	Toxicokinetic study
				Females	Females
1	Vehicle 1	0	0.1	10	3
2	Vehicle 2	0	0.1	10	3
3	GRFT (BS-derived)	3	0.1	10	9
4	GRFT (BS-derived)	5	0.1	10	9
5	GRFT (NB-derived)	5	0.1	10	9

^*a*^ BS, *Bacillus subtilis*; NB, *Nicotiana benthamiana*.

Mortality, clinical signs, body weight, food consumption, clinical pathology, toxicokinetics, ADA in serum, organ weights, macroscopic pathology, and histopathology were evaluated.

Blood samples (approximately 0.5 mL) for ADA analysis were collected from main study animals pre-treatment and on day 29. Serum samples were stored at −70°C prior to analysis.

### Clinical pathology

Animals were not fasted prior to blood collection. Blood was collected on the day of necropsy for hematology and clinical chemistry evaluation and was collected for coagulation parameters at the time of euthanasia.

### Toxicokinetics

Blood samples (approximately 0.5 mL) for toxicokinetic analysis were collected from animals assigned to the toxicokinetic satellite groups at pre-dose and 1, 2, 4, 8, 12, and 24 h after dosing on days 1 and 26. Serum samples were stored at a target temperature of −70°C prior to analysis.

### GRFT assay in rat serum

Samples were analyzed at KCAS Bio using the MSD-ECL assay. Briefly, a plate was coated and incubated at room temperature with Bt-anti-QGRFT mAb. Following incubation, the plate was washed to remove excess coating reagent and blocked with Pierce Protein Free buffer to prevent non-specific binding. After incubation, the plate was washed, and standards, QCs, and study samples were diluted 1:100 in buffer prior to being added to the assay plate. After sample incubation at room temperature, the plate was again washed, and a rabbit anti-QGRFT pAb detection antibody was added to the plate. After primary detection antibody incubation at room temperature, the plate was again washed to remove any unbound material, and a Sulfo-Tag goat anti-rabbit IgG detection antibody was added to the plate. The assay was incubated again at room temperature before undergoing a final wash step. A 2× Read T Buffer was added, and the MSD plate was read using Methodical Mind software. Sample concentrations of GRFT were interpolated from a standard curve generated with known concentrations of GRFT. This method was previously validated over the range of 10.0 to 5,000 ng/mL. Data were collected using Meso Sector S 600 utilizing acquisition software Methodical Mind v1.0.38. The processing and data management software used was Watson LIMS version 7.5 SP1.

Since GRFT was undetectable in all samples collected, the planned toxicokinetic parameter estimation by non-compartmental analysis could not be completed.

### Anti-drug antibody assay in rat serum

ADA concentrations in rat serum were analyzed using the MSD-ECL assay at KCAS Bio. Detection of the anti-drug antibodies was done using a screening approach with a cut-point established to support a 1% false-positive rate. In the screening assay, positive control and unknown samples are diluted 1:10 in 300 mM acetic acid and incubated for 10 to 20 min. At the end of incubation, a 2× stock of neutralizing Master Mix (Biotinylated GRFT and Ruthenylated GRFT, KCAS) was added in equal volume to the acidified samples and incubated for approximately 1 h to allow for immune complex formation. At the end of this master mix incubation, samples were transferred to a blocked MSD Streptavidin plate and incubated for approximately 1 h. Finally, the MSD Streptavidin assay plate was washed with 1× PBS-T, then 2× MSD Read Buffer was added, and the plate was read promptly on an MSD Sector Imager 600. If the RLU response was greater than the screening cut point, then the sample was reported as positive for anti-GRFT antibodies.

### Terminal procedures

At the end of the treatment period, rats were euthanized by carbon dioxide inhalation, followed by an appropriate secondary method. A complete gross pathological examination was performed, including evaluation of the carcass, all external surfaces and orifices, the cranial cavity and external surfaces of the brain, and the thoracic, abdominal, and pelvic cavities with their associated organs and tissues. Organ weights were recorded, and representative samples of tissues were collected and preserved in 10% neutral buffered formalin. Hematoxylin and eosin-stained paraffin sections were prepared for the vagina, cervix, oviduct, and gross lesions from all main study animals, and a detailed microscopic evaluation was performed.

## SUPPLEMENTAL MATERIAL

Supplemental figures spectrum.01966-25-s0001.pdf Fig. S1 and S2.

ASM does not own the copyrights to Supplemental Material that may be linked to, or accessed through, an article. The authors have granted ASM a non-exclusive, world-wide license to publish the Supplemental Material files. Please contact the corresponding author directly for reuse.
