# Supplementary material for: Preclinical evaluation of high-dose Griffithsin carrageenan fast-dissolving insert for HIV, HSV-2, and HPV prevention
Source: Microbiol Spectr. 2026 Apr 20;14(6):e01966-25. doi: 10.1128/spectrum.01966-25 (PMC13228069; doi:10.1128/spectrum.01966-25)
Supplement: Supplemental figures — Fig. S1 and S2. [file spectrum.01966-25-s0001.pdf]

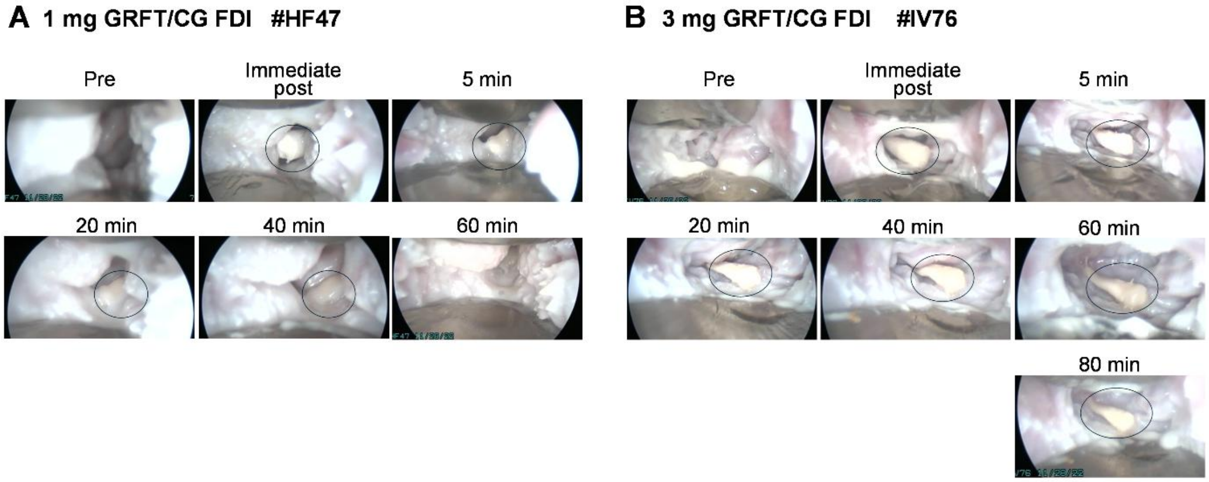

Supplemental Figure 1. Examples of colposcopy images from macaques administered 1 mg or 3 mg GRFT/CG FDIs (pre- and post-insertion).

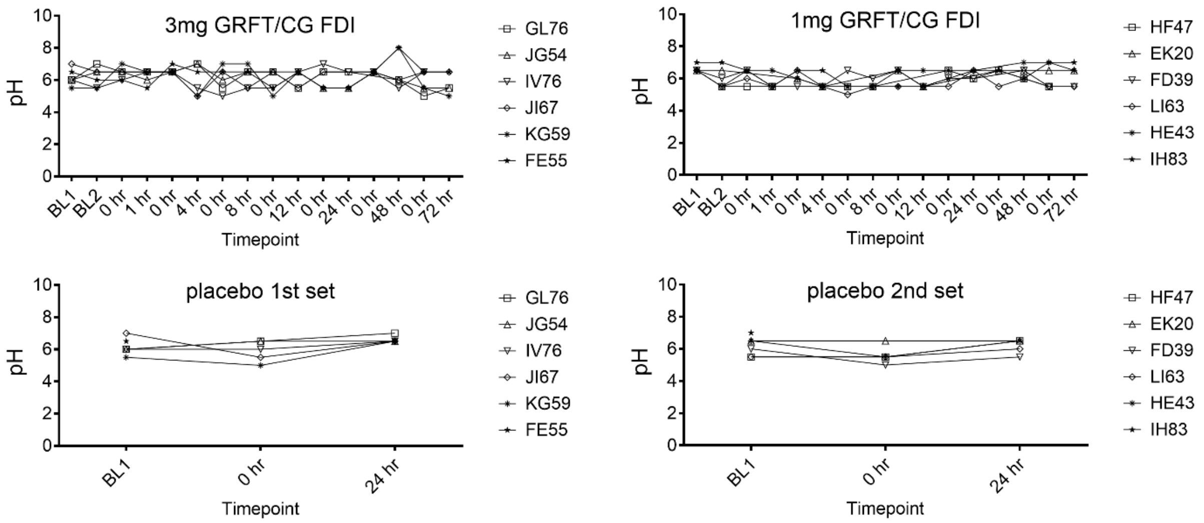

Supplemental Figure 2. **Vaginal pH**. pH was measured at baselines (BLs), immediately before FDI insertion (0 hr), and at the indicated timepoints. Each symbol indicates an individual animal.
